# Decision-making on the fly: a qualitative study of physicians in out-of-hospital emergency medical services

**DOI:** 10.1186/s12873-023-00830-w

**Published:** 2023-06-07

**Authors:** Ema Karmelić, Henrik Lindlöf, Jamie Linnea Luckhaus, Moa Malmqvist Castillo, Veronica Vicente, Karin Pukk Härenstam, Carl Savage

**Affiliations:** 1 Medical Management Centre, Department of Learning, Informatics, Management and Ethics, Karolinska Institutet, Tomtebodavägen, Stockholm, 18A 171 77 Sweden; 2 Department of Clinical Science and Education, Karolinska Institutet, Södersjukhuset, Stockholm, Sweden; 3 The ambulance medical service of Region Västmanland, Västerås, Sweden; 4 The ambulance medical service in Stockholm (AISAB), Stockholm, Sweden; 5 Academic EMS, Stockholm, Sweden; 6 Department of Womens and Childrens Health, Karolinska Institutet, Stockholm, Sweden; 7 School of Health and Welfare, Halmstad University, Halmstad, Sweden

**Keywords:** Pre-hospital, Guidelines, Healthcare management, Clinical judgment, Ambulance services, Patient safety

## Abstract

**Background:**

Out-of-hospital Emergency Medical Services (OHEMS) require fast and accurate assessment of patients and efficient clinical judgment in the face of uncertainty and ambiguity. Guidelines and protocols can support staff in these situations, but there is significant variability in their use. Therefore, the aim of this study was to increase our understanding of physician decision-making in OHEMS, in particular, to characterize the types of decisions made and to explore potential facilitating and hindering factors.

**Methods:**

Qualitative interview study of 21 physicians in a large, publicly-owned and operated OHEMS in Croatia. Data was subjected to an inductive content analysis.

**Results:**

Physicians (mostly young, female, and early in their career), made three decisions (transport, treat, and if yes on either, how) after an initial patient assessment. Decisions were influenced by patient needs, but to a greater extent by factors related to themselves and patients (microsystem), their organization (mesosystem), and the larger health system (macrosystem). This generated a high variability in quality and outcomes. Participants desired support through further training, improved guidelines, formalized feedback, supportive management, and health system process redesign to better coordinate and align care across organizational boundaries.

**Conclusions:**

The three decisions were made complex by contextual factors that largely lay outside physician control at the mesosystem level. However, physicians still took personal responsibility for concerns more suitably addressed at the organizational level. This negatively impacted care quality and staff well-being. If managers instead adopt a learning orientation, the path from novice to expert physician could be more ably supported through organizational demands and practices aligned with real-world practice. Questions remain on how managers can better support the learning needed to improve quality, safety, and physicians’ journey from novice to expert.

**Supplementary Information:**

The online version contains supplementary material available at 10.1186/s12873-023-00830-w.

## Introduction

Emergency Medical Services (EMS) require fast and accurate on-site assessment of patients that present with varied and unique medical, personal, and environmental circumstances [1]. This necessitates resourcefulness and flexibility in clinical judgment regarding immediate patient care as well as multiple operational and resource-management decisions made under pressure and based on limited information [2]. The level of clinical judgment required is partly determined by the EMS model employed. Whereas the Anglo-American “scoop-and-run” pre-hospital EMS (PEMS) paramedic-based approach limits in-field treatment and intervention, the Franco-German out-of-hospital EMS (OHEMS) approach employs a “stay-and-stabilize” strategy that often requires emergency physician support [3].

Working in a context that is volatile, uncertain, complex, and ambiguous is inherently risky [4]. Incorrect decisions may lead to medical errors and adverse events, which present a threat to clinical outcomes and both patient and provider safety [5]. It is clear that professional training alone is not enough to mitigate the risks, which has led to the application of team-training and decision-support tools, e.g. checklists, guidelines, protocols, and standard operating procedures (SOPs) in health care [6, 7]. However, there is a high variation (7.8–95%) in their use [8] such that protocols with proven effectiveness are not applied, while practices proven to be less effective and even potentially harmful persist [9]. Guideline compliance in prehospital settings is influenced by multiple factors, including perceptions of their quality and value, practitioners’ age, experience, and willingness to take risks [10], unique patient and organizational circumstances [8], and poor strategies for creating and disseminating guidelines [9]. In light of these insights, there is a need for research that identifies risks associated with patient care decisions in OHEMS settings and what influences adherence to guidelines or implementation strategies that improve adherence [11, 12].

Research on decision-making in OHEMS settings has primarily focused on paramedics, nurses, or a combination of EMS professionals in the context of: resuscitation [13–15], conveyance [2, 16–19], triage [20, 21], ethical issues [22, 23], and clinical judgment [1, 24, 25]. While exploring decision-making from different angles, these studies consistently suggest that the complexity of such highly contextual and multifactorial work processes require a reassessment of education and training programs, guidelines, and feedback mechanisms to improve performance and patient safety. Moreover, the continuous growth in demand and complexity of patient conditions, which reflect the needs of an aging society and chronic and palliative patients, can lead to questions about the capability of paramedic-led teams and calls to explore alternative care pathways and reevaluate EMS design [17, 26, 27].

There is less research on physician decision-making in OHEMS, which has mainly studied resuscitation [28, 29], care of palliative patients [30, 31], duration of interventions [32], and the impact of cognitive and non-technical skills on care quality [33, 34]. As physicians receive a broader education and acquire a wider set of skills and competencies, there is a greater decision-making scope. In addition, given the responsibility they bear for their patients, it is important to better understand the types of decisions they make, the kind of support that they need beyond existing guidelines, and the implications for patient safety. Therefore, the aim of this study was to increase our understanding of physician decision-making in out-of-hospital emergency medical services, to characterize the types of decisions made, and to explore potential facilitating and hindering factors.

## Methods

### Study design

We chose a qualitative approach as it was deemed the most appropriate to gain in-depth insights into personal lived experiences, behaviors, and perspectives [35], in a context where shame-and-blame of individual physicians is a commonplace cultural norm [36]. We report in accordance with the Consolidated Criteria for Reporting Qualitative Studies (COREQ) checklist (Appendix 1) [37].

### Study setting

We conducted the study at a large regional OHEMS organization in Croatia. In 2021, the organization served a population of 769,944, employed 241 ambulance staff, answered 150,000 phone calls, and conducted 75,714 field interventions, of which 83% were of low acuity.

Croatian OHEMS are offered as a publicly owned, financed, and operated regional public service, coordinated by the Croatian Institute for Emergency Medicine [38]. The Institute provides guidelines, algorithms, and procedural suggestions for medical conditions [39], most often not tailored to the specific context of operation (Appendix 2). OHEMS provide medical interventions and procedures 24/7 in out-of-hospital settings and during ambulance transport [39]. Ambulance teams are characterized as either “Team 1” (3 members: physician, nurse, and a driver) or the more recent “Team 2” (2 members: 2 nurses; one of them drives), which was introduced due to a physician shortage [38]. The choice of which team to dispatch is dependent on an assessment of which competencies are required. Physician area of responsibility includes a physical exam, diagnosis, treatment selection, certain procedures, and team coordination. Nurses assist with the physical exam and carry out diagnostic and treatment procedures. The driver is responsible for safe transport and can assist others when needed [39]. It is common for physicians at the start of their career to work in OHEMS [38]. After about 1 year of ambulance work, physicians usually work at a dispatch center a few shifts per month. Dispatch centers follow the Croatian adaptation of the Norwegian Index for Medical Emergency Assistance [39].

### Study participants

We chose participants using purposive sampling to ensure the requisite experience (1-year was deemed sufficient for insight into work-as-done) as well as interest and ability to participate in the study [40]. We contacted physicians via email and strove to ensure that participants reflected the overall physician population in the organization in terms of gender, age, and work experience.

### Data collection

We initially tested a semi-structured interview guide with three interviews. After analyzing the interview transcripts, which lacked a smooth flow, we opted to use an open interview approach with a single query statement: *“I would like you to tell me about your compliance to guidelines in your decision-making around patients’ treatment.”* The query statement was designed as a prompt to stimulate a discussion about physician decision-making. It was based on the assumption, grounded in the emergency medical practice experience of the research team, that guidelines are often the point of departure for decision-making, and that if this was not the case, it would regardless spur a discussion. In this context, the term “treatment” was used to refer to the entire patient pathway from receiving a call to the conclusion of the intervention. To ensure that we collected data that answered our research question and collected specific and concrete examples, we posed two follow-up questions when needed: *“What were the consequences for your patient?”* and *“What additional support, if any, would you have liked to receive?”.*

We piloted this revised approach twice. Since the flow of the interview transcripts improved, and no additional changes were made, these pilot interviews were included in the analysis. Individual interviews were conducted by EK (female with training in qualitative research) in Croatian, during February-March 2022 in a private area of participants’ choosing, often their workplace. Interviews lasted approximately one hour and were digitally recorded. Data collection continued until theoretical saturation, i.e. when further data collection was deemed superfluous as no fundamentally new information surfaced regarding common views held by participants [41]. EK transcribed the interviews *verbatim*.

### Data analysis

We utilized an inductive content analysis, where categories were derived from the raw data, as it is a content-sensitive method applicable where there is little or no *a priori* understanding or knowledge about the phenomenon of interest due to the lack of published research [42]. EK (as the native Croatian speaker) read through the transcripts repeatedly to develop familiarity with the data and identified meaning units that captured key thoughts using NVivo qualitative data analysis software; QRS International Pty Ltd. Version 12, 2018. These were condensed into codes in English by EK and then grouped into general themes and categories using the Miro visual collaboration platform (www.miro.com). To improve trustworthiness, each category and its constituent codes were reviewed by three authors. Discrepancies were discussed until consensus was achieved. The findings section was shared with all participants. Seven chose to respond and validated the findings, i.e. they felt their experience was captured in a fair and correct manner.

### Ethical considerations

Participants provided their informed consent prior to commencement of the interviews after receiving written and oral information about the study. We explained we would strive to pseudonymize the results, especially considering the delicate and confidential nature of the topic. The study was vetted and approved by the Ethical Board of the Institute of Emergency medicine of the City of Zagreb (nr. 892).

## Results

Of the twenty-one physicians interviewed, the majority were young, female, with less than three years of experience, and no specialist training (Table 1). Four physicians who had agreed to interviews later declined because of scheduling issues.

**Table 1:** Participant characteristics

Characteristics	Participants (n = 21)
Age	
25–29 years	12
30–39 years	3
> 50 years	6
**Gender**	
Female	15
Male	6
**Length of work**	
< 3 years	12
5–10 years	3
> 20 years	6
**Professional title**	
Medical doctor	16
Medical doctor, specialist in emergency medicine	5

We identified four themes related to the types of decisions made, influential factors, perceived implications on quality of work and patient care, as well as the desired support for decision-making (Table 2).

**Table 2:** Themes, categories, and sub-categories with illustrative quotations

Theme	Category	Sub-categories	Illustrative quotation
1. Types of decisions made	1.1. Transport?	Transport; Leave patient at scene	“I started taking more patients to the hospital because it’s impossible to make a proper assessment with the diagnostic tools we have.” (P2)
	1.2. Treatment?	Administer treatment; Do not administer treatment	“For some conditions, those ‘half-conditions’, where I can’t be sure what’s happening without more diagnostics… I don’t know what to do with those patients. I would rather just transport them to the hospital for further diagnostic tests as I feel giving them therapy might do more harm than good.” (P1)
	1.3. How to transport and/or treat?	Choice of transport and/or treatment	“Sometimes my decision depends on how far away we are from the hospital. Is it more important to bring the patient to hospital as fast as we can so he can get full diagnostics and treatment, or to spend 10 more minutes on the field to follow all the steps from the guidelines?” (P7)
2. Factors that influenced decision-making	2.1. Physician-related	Learning journey; Emotions; Fatigue, burnout, and suicide; Personal values	“It’s all nice when we are relaxed and we go to work well-rested, no frustrations, then we are more objective. But when we have all these other things…when we are depressed, frustrated, we don’t sleep well, we are not satisfied…then we react abruptly, communication is worse, negative energy is created between patients and us, and then the treatment of the patient suffers.” (P17)
	2.2. Patient-related	False statements; Violent behavior; Complaints and lawsuits	“Lawsuits are not as common as complaints to our employer…they happen often. Yes, people like to report us. But it’s not only reporting, there are also threats and violence, verbal and physical. We find ourselves in very uncomfortable situations. It’s enough if a doctor loses his license once to suffer the consequences for the rest of his life. It’s what we all fear. We feel very exposed, and we make decisions based on that fear.” (P7)
	2.3. Organization-related	Training; Feedback; Diagnostic and treatment tools; Guidelines; Team dynamic; Relationship with management	“My experience doesn’t mean much if I keep doing the same thing without knowing if it’s the right or wrong thing to do… I can keep repeating mistakes if nobody tells me that it led to a bad outcome for the patient… Feedback is important to build my experience and to use for further work.” (P21) “It would be ideal if leadership would spend some time in the field… I think it would be easier if they could identify with workers on the field, if they would understand them…working conditions would be better for sure…they would show more empathy towards difficulties workers go through and treat them better.” (P12)
	2.4. Health care system-related	“Grey-zone” situations; Emergency services overcrowding; Tensions at patient handovers	“Given that OHEMS should not really deal with this, as it should be covered by GP’s and palliative team house visits, there are no clear guidelines for us.” (P15)
3. Perceived quality of work and patient care	3.1. Variability in decision-making	Absence of diagnostic and treatment tools; Lack of clear guidelines and work protocols; Inadequate feedback and quality control mechanisms; High turnover rate	“There shouldn’t be such variability in our work because it influences the quality of care. Patients should not be treated differently depending on who comes to their house.” (P21)
	3.2. Medical errors	Malfunctioning equipment; Untimely care; Unnecessary treatment	“Increased demand leads to overcrowding the system, which leads to fatigue and slows down the work, so some real emergencies might wait too long, and increase patient mortality as a result.” (P20)
4. Decision-making support desired	4.1. Organization-related	Training; Feedback; Guidelines; Team dynamic; Relationship with management	“The Institute could publish their own revised and modified guidelines, depending on the available equipment.” (Participant 14)
	4.2. Health care system-related	Primary healthcare reform; Health literacy; Work environment; Coordination between different public services	“There should be a system that would connect hospital, OHEMS, and family medicine… because elderly patients mostly can’t tell you which medications they are taking, so it would be easier to check that information in the system. Also, it would be good to know if you made the right decisions and what happened to the patients after you took them to the hospital. That would give us moral satisfaction and would help us learn.” (P8)

### Theme 1. Types of decisions made

Participants described a highly variable work process that involved patient assessment (including measuring vital signs, preforming a physical examination, taking a medical history, evaluating patients’ symptoms, and developing an overall impression of patients’ environment) and decision-making, which revolved around three main questions:

- Whether or not to transport patients to the hospital?
- Whether or not to treat?
- If yes to either, then how?

Participants explained that these decisions required weighing the perceived risks of each option for each patient.

### Theme 2. Factors that influenced decision-making

Participants described how each question often became complex and unclear due to physician-, patient-, organization-, and healthcare system-related factors, which influenced their decision-making process and guideline compliance.

### Physician-related factors

Participants reflected on the learning journey physicians make from “novice” to “expert”: a seasoned physician accrues knowledge through first-hand experiences and by observing colleagues, whereas a novice lacks practical and applied knowledge. Guidelines were therefore especially valued by novices. However, guidelines presumed ideal working conditions and were therefore often not applicable. Many reported how they instead learned through “trial and error” and developed their own personal “guidelines”.

Participants described work as stressful, working conditions as poor, and expressed feeling undervalued and underpaid. They explained how feeling tired and overwhelmed had a negative impact on their relationship with and attitude towards patients, made them prone to conflicts or acquiescent to patients’ demands, and could lead to burnout. Some participants reflected upon co-workers’ suicides, which they thought might have been connected to high levels of stress and exhaustion and a lack of psychological support.

### Patient-related factors

The severity of the patient’s condition was the paramount factor. “Real” emergencies were often easily identifiable due to training and guidelines, and it was easier to make decisions. Factors that increased complexity were patients’ socioeconomic situation, which influenced care quality at home. Participants also described that patients could lie or misrepresent their symptoms when calling emergency services. This could lead physicians to lose trust and empathy for their patients and consequently risk an underestimation of the severity of health issues. Physicians saw an increasing trend of verbal and physical aggression, complaints, and lawsuits, which they increasingly answered by practicing “defensive medicine”, e.g. “giving in” to patient demands to avoid conflict; even administering unnecessary therapies merely to satisfy patients.

### Organizational factors

Organizational factors included training and feedback, diagnostic tools, guidelines, team dynamics, and physicians’ relationship with management.

#### Training and feedback

Participants’ views of their organization’s training courses differed widely. Some saw them as useful, while others felt they were too brief and irrelevant to real-world experiences, especially in terms of equipment and non-urgent situations. The consensus was that management was not investing enough in staff education.

Structured performance feedback was highly valued, but seldom received. Participants described how they sought information on patient outcomes from hospital staff, complaint logs, and occasionally managers. This was described as time-consuming, impractical, and more importantly – illegal – but nevertheless deemed necessary to improve decision-making.

#### Diagnostic and treatment tools

Participants expressed their frustration about daily problems related to the limited availability or malfunction of important diagnostic and treatment tools (e.g. defibrillator/ECG, glucometer, thermometer, paediatric pulse oximeter, infusion heaters, equipment for intubation, or lack of adult intraosseous device, Foley catheters, nasogastric tube, medication for rapid sequence intubation, insulin, etc.). In the absence of objectively measured patient parameters, decision-making depended on subjective assessments and improvisation. Aware of how this increased the risk for medical error, most participants preferred transporting patients to the hospital for a more thorough examination, especially pregnant women, children, and patients with chest pain.

#### Guidelines

Guidelines were seen as a useful tool for decision-making and to standardize care to improve patient outcomes and mitigate the risk for medical errors. However, despite access to multiple sources of guidelines, participants acknowledged compliance issues, including lack of organizational policies on which ones to use, where to find them, or how to apply them. Instead, there were “unwritten rules”, i.e. internal and unofficial instructions and expectations on how to act in certain situations. This knowledge was imparted by senior colleagues. The lack of clarity was faulted for mistakes.

Most participants believed it impossible to create situation-specific guidelines, as each patient has a unique set of symptoms and socioeconomic factors to be considered. Furthermore, when feeling like the patient’s or their own safety was compromised, they did not follow guidelines to the letter. This mostly included traffic accidents and interventions involving psychiatric or violent patients.

Some participants felt obliged to adhere to guidelines as it gave them a sense of legal protection should anything go wrong. Others felt the guidelines would not protect them in court, as they perceived them more as recommendations rather than legally binding. Lastly, all participants emphasized how insufficient and malfunctioning diagnostic and treatment tools prevented them from following the guidelines and providing the best patient care.

Most participants had attended the mandatory training for the OHEMS dispatch center. All admitted to not using the official dispatching guidelines (Croatian adaptation of the Norwegian Index), instead relying on personal judgment. This appeared to be encouraged by management, who had “unwritten rules” for triaging patients more suited to local needs.

#### Team dynamics

Close and friendly relationships and a positive atmosphere within the team were seen as important for guideline compliance and crucial for care quality. However, variation in experience levels combined with a lack of clear role descriptions led to confusion and poor coordination. Since most physicians were younger women and the drivers and nurses often older men, patients sometimes assumed the latter were physicians, which made establishing authority difficult. Some drivers and nurses interfered in decision-making and pressured younger doctors by telling them that “good doctors leave patients at home; bad doctors drive everyone to the hospital.” Worried about their image, some participants felt they had to balance patients’ interests with avoiding team tensions.

#### Relationship with management

Participants experienced a lack of support from management who they universally expressed “did not have my back”. Official reports on faulty equipment or vehicles seldom garnered a response. Management sometimes criticized physicians for following guidelines. Psychological and legal support, especially in the context of patient complaints, lawsuits, and acts of violence, was missing.

### Healthcare system-related factors

Complexity of patient care conditions, ethical dilemmas, value judgments, and lack of patient trajectory planning on a healthcare system level resulted in “grey-zones” that had increased in frequency in recent years. These included palliative/terminally ill patients, or elderly patients often with multiple comorbidities, and often in nursing homes. There were also patients without life-threatening conditions, but which could become so if not recognized or treated in time. Without clear guidelines, it was challenging to decide on transport and treatment.

Grey-zone situations could start out seemingly simple, but become complex due to a lack of coordination across organizational boundaries, such as with law enforcement, social services, or other healthcare providers (e.g. primary care or hospital departments).

Emergency department overcrowding could induce physicians to avoid transporting patients due to the potentially greater risk incurred by long waiting times, e.g. for elderly patients during the Covid-19 pandemic. Already inadequate resources were further strained by numerous non-acute calls from citizens, who often could not differentiate between urgent and non-urgent conditions.

Additionally, there was a certain animosity between out-of-hospital and hospital-based physicians caused by overwhelming work conditions and differences in working principles and treatment preferences. This could lead to disputes during patient handovers, so participants sometimes preferred abdicating treatment choices to hospital-based physicians.

### Theme 3. Perceived quality of work and patient care

Participants emphasized the importance their decision-making, and the factors that influenced it, had on the quality of their work and patient outcomes. They described a high variation in decision-making due to a lack of standardization in their work environment: malfunctioning equipment, absence of diagnostic and treatment tools, lack of clear guidelines and work protocols, and inadequate feedback and quality control mechanisms. High staff turnover rates led to a constant influx of new inexperienced doctors that hurt guideline compliance and team dynamics. Participants felt that these factors increased the risk for medical errors, including the loss of valuable time, which could make it difficult to provide care within the “golden hour”.

### Theme 4. Decision-making support

Participants made numerous suggestions and recommendations about how to facilitate decision-making processes.

### Organization-related recommendations

Training and formalized structured feedback were highlighted as areas for improvement. Participants desired annual and standardized training courses with modules on communication and case studies, an official mentorship program, and telemedicine connections between new physicians and experienced colleagues. A centralized information technology (IT) system could provide information on patients’ medical history to facilitate assessment and decision-making. Officially authorized feedback on patient outcomes and quality control mechanisms for continuous monitoring of work processes and outcomes were strategies suggested to identify areas for individual and organizational improvement. Team-building activities were desired as team relationships and a positive atmosphere were seen as crucial. A clear description of duties and responsibilities would ensure everyone had the requisite competencies and improve team coordination.

Participants asked for improved guidelines, ideally developed by managers, tailored to the work context (equipment, medications available, and specific challenges and changes within the local healthcare system), and encompassing the wider span of clinical and administrative tasks encountered in everyday work. Participants also wished for standardized and updated equipment as well as a response from managers on issues brought up in staff field reports, such as faulty equipment.

Participants emphasized the need for psychological support, such as professional psychological services, routines for after-action debriefing, or shortened shifts after especially taxing interventions (e.g. resuscitations). Due to the perceived high risk of lawsuits, participants saw a need for legal counselling and sharing liability with managers.

### Healthcare system-related recommendations

Participants felt improved health literacy and health education for the public could lead to a decrease in the number of non-urgent calls and improve communication with patients. A separate phone line for medical advice in non-acute situations was also suggested, as well as improved coordination with law enforcement, social services, and other healthcare actors.

## Discussion

We sought to understand physician decision-making in out-of-hospital emergency services. We found that physicians made three basic decisions. The first two were binary (whether or not to transport or to treat). The third was related to how – how to transport and how to treat. Typically, binary (yes/no) questions related to *if* are seen as simpler and questions related to *how* are more complex [43]. However, all three decisions were made more complex and perhaps unduly influenced by factors at the micro-, meso-, and macrosystem levels that forced physicians to make impromptu decisions *on the fly* and generated a high degree of variability that increased the risk for medical errors. Several of the factors that contributed to this complexity are corroborated in the literature: time pressure, patient condition, work environment, experience and competence level, team collaboration, and organizational support [2, 17, 44]. All these factors have been found to influence decision-making processes in different contexts. The educational backgrounds of ambulance staff, where lack of team and organizational support, adequate training and feedback, and ambiguous patient conditions and environment further increased the difficulty of making prompt and qualified decisions.

At the clinical micro-system level, where the patient and provider team meet, the findings suggest physician experience and competency level are fundamental. Most participants were women and at the start of their medical careers. This group may be more susceptible to internalizing failure with a subsequently higher rate of imposter phenomenon, depression, and suicide compared to other populations of academics [45, 46]. As gender roles in this setting were opposite the traditional stereotypes of men as doctors and women as nurses, female physicians often experienced an implicit bias from both patients and team members. Novice female physicians struggled to establish authority when coupled with older and more experienced male nurses and drivers, which sometimes complicated work. For the novice yet to develop “expert” tacit knowledge [47], a learning orientation supported by guidelines, checklists, SOPs, training, feedback, and mentors is essential to progress in expertise and develop the requisite competencies and capabilities [48, 49]. The organizational context can support this transition, for example high reliability organizations standardize routines and capture these in protocols and checklists [50].

Participants described meso-level constraints related to the availability of medication, equipment, vehicles, medical and legal competency levels, personal values and ethics, patient interaction, and work engagement levels which influenced guideline compliance, and created situations that required non-linear thought processes based on experience and instinct. To support guideline use, they need to be aligned with the natural decision-making process that allows for adaptation to changes in patient condition and surroundings [1, 20], or strategies explicated for how to address the needs of “grey zone” patients [10], which do not fit existing protocols. Indeed, while participants expressed a need for a more explicitly delineated structure, their adeptness to create and follow unwritten rules opens space for exploring an important concept in patient safety of facilitating flexibility of decision-making and embracing work complexity [51]. This provides additional support for not only teaching competencies, but also educating for capabilities [49].

Effective training for novices focuses on the development of practical, technical, and communication skills with observed benefits associated with performance in the field and confidence levels [17, 32]. Feedback is an effective quality improvement strategy that can improve clinical performance and patient outcomes [52]. Constructive, mutually respecting feedback from hospital staff can improve protocol compliance and decision-making on patient treatment in EMS [53]. Quantitative data analysis, e.g. statistical process control, integrated into governance systems can close feedback loops and lead to adjustments in practices that improve patient outcomes [54]. Feedback using IT-systems, such as real-time telemedicine support are useful – telemedicine consultations with emergency physicians by ambulance staff can improve efficiency and safety in assessment and transport of high-risk patients [55], stabilize life-threatening conditions [56], and decrease unnecessary transports of non-urgent cases [19]. And case studies, as raised by participants, support learning from concrete experiences [48, 57].

Sound meso-system leadership and management practices that support the development of and reinforce key learning practices are of vital importance in the volatile, uncertain, complex, and ambiguous context that characterizes OHEMS. Participants described how the work environment, with its lack of support for learning (guidelines, SOPs, training) and well-being (psychological and legal support, communication) created a feeling that the organization “did not have their back” and they were on their own. This negatively affected their feelings of psychological safety as they were afraid of reprisals and losing their license, which they felt stymied quality and safety improvement efforts, a pattern that has also been described in recent medical literature [2, 17]. The burden of responsibility participants carried and internalized was increased by the threat of legal repercussions, the lack of support structures, and continual exposure to questioning from patients, nurses, managers, and themselves. Participants’ descriptions of increased threats, violent behavior, and litigation create fear, lower job satisfaction and decrease motivation, which can negatively impact the provision of care [58, 59] and create space for medical errors, making them susceptible to developing a second victim phenomenon [60]. The coping strategy participants described – to practice defensive medicine and acquiesce to demands even though they violated guidelines or were without medical merit has, in hospital settings, led to decision-making that has not been in patients’ best interests [61, 62].

Medical settings that foster psychological safety and a positive learning environment, are better equipped to learn from mistakes and improve patient safety and employee well-being [63]. Team training, such as Crew Resource Management, can also contribute to a learning culture by improving team dynamics, communication, and a safe atmosphere [7, 64]. These techniques can also be paired with simulators to promote training under realistic conditions and help learners to deal with the aftermath and consequences of failed interventions [13]. More research is needed on strategies for how managers can lead to better support staff in OHEMS.

In the macrosystem, participants described issues related to coordination over organizational boundaries (e.g. contractual demands from law enforcement and process bottlenecks caused by emergency department overcrowding) and demographic changes in patient population. Campaigns to improve health literacy could reduce “overuse” of emergency services [65]. Reducing emergency overcrowding could reduce treatment delays, mortality, medical errors, and improve patient satisfaction [66]. Demographic changes have led to a shift towards older patients and more chronic conditions with an increase in low-acuity and non-urgent cases [2, 17, 26]. These are often more clinically complex to manage. Treatment outcomes could benefit from care-process redesign across organizational boundaries [67, 68] or innovative approaches to primary care [69].

### Limitations/Methodological considerations

We found negative undertones in the data and analysis. The first author was previously employed at the study setting and had worked with several of the study participants, which we think contributed to a high level of trust, openness, and honesty during the interviews, vital when exploring a sensitive topic such as this one [70]. We did realize that our opening interview question could be an expression of our own bias of the importance of guidelines in emergency care. We made efforts to develop reflexivity by identifying and discussing any *a priori* bias, such as opinions on OHEMS and the employer in question, own experiences and difficult situations, views of the different professions, as well as more general prejudices or learned behaviors and perspectives that could bias data collection, analysis, and the reporting of the results [71]. We therefore feel confident in interpreting the results as another example of the importance of considering staff well-being and engagement as one of the four aims of health care [72].

We tried to improve transferability by providing a more detailed contextual description as a point of departure for the subsequent analysis and findings. Trustworthiness of the findings was increased due to the *a priori* understanding of the context, which was helpful in the analysis, and the rigor of the analysis process itself, which involved three additional researchers with backgrounds in public health, medicine, nursing, and research in medical management. Authenticity was enhanced through a participant group that reflected the gender, age, professional development, and work experience of physicians working in the setting.

## Conclusion

Physician decision-making in out-of-hospital emergency medical services involves three deceptively simple decisions on transport, treatment, and how to carry out each. Physicians experienced these decisions as (unnecessarily) complex due to contextual factors at the micro, macro, and particularly meso system levels. We found that the burden of responsibility for answering these questions, and for the answers themselves, was borne by individual physicians, often at an early phase of their medical career. In itself, this could create quality and safety issues related to patient experience, patient outcomes, and staff well-being. However, factors outside traditional medical training curricula had a potentially undue influence on decision-making. Responsibility for the meso level is the province of managers. They have the means to create and support a safer learning environment by developing clear guidelines and standardized operating procedures aligned with real-world decision-making processes, strategies for when guidelines do not fit such as real-time tele-medicine support from older colleagues, reflective feedback, and psychological safety. These are ingredients not only important for organizational learning and safety, but also necessary to support physicians’ journey from novice to expert. There is a clear need for further research on how management and leadership strategies can better support staff as they make decisions-on-the-fly in out-of-hospital emergency medical services. With more effective support from employers and awareness of the contextual factors that influence decision-making beyond the patient themselves, staff will be able to feel better, learn better, and do better.

## Electronic supplementary material

Below is the link to the electronic supplementary material.

Supplementary Material 1

Supplementary Material 2

## Data Availability

The datasets generated during the analyses of the current study are available from the corresponding author on reasonable request.

## Abbreviations

COREQ: Consolidated Criteria for Reporting Qualitative Studies
ECG: Electrocardiogram
EMS: Emergency Medical Services
GP: General Practitioner
IT: Information Technology
OHEMS: Out-of-hospital Emergency Medical Services
PEMS: Pre-hospital Emergency Medical Services
SOP: Standard Operating Procedure
