# Supplementary material for: Decision-making on the fly: a qualitative study of physicians in out-of-hospital emergency medical services
Source: BMC Emerg Med. 2023 Jun 7;23:65. doi: 10.1186/s12873-023-00830-w (PMC10246870; doi:10.1186/s12873-023-00830-w)
Supplement: Supplementary file 2 — Supplementary Material 2 [file 12873_2023_830_MOESM2_ESM.docx]

Appendix 2

**List of guidelines used in the Croation EMS**

The Croatian Institute for Emergency Medicine issues standard operating procedures, protocols, algorithms, and educational programs, that should be followed by all members of the ambulance team (Ordinance on Conditions, Organization, and Manner of Provision of Emergency Medicine (OG 71/16)).

There are four guideline publications found on the official website of the Croatian Institute for Emergency Medicine (<https://www.hzhm.hr/strucne-publikacije/smjernice>). The main procedures, protocols, and algorithms are published in the 380-page textbook, *Guidelines for work in out-of-hospital emergency services* (Bošan & Majhen, 2012). It covers the following chapters:

1. Emergencies in adults (examination, abdominal pain, altered level of consciousness, dyspnea, headache, psychiatric disorders, non-traumatic chest pain, pain management)
2. Specific treatment methods (acute coronary syndrome, anaphylaxis/allergic reactions, asthma, chronic obstructive pulmonary disease, pulmonary embolism, stroke/transient ischemic attack, convulsions, gastrointestinal bleeding, emergency glycemic conditions, drowning, hyperventilation syndrome, heat stroke, hypothermia, meningococcal sepsis, overdose, and intoxication)
3. Adult life support (basic life support, advanced life support, foreign body airway obstruction, implantable cardioverter-defibrillators, cardiac rhythm disorders, dying)
4. Adult trauma emergencies (examination, abdominal trauma, head trauma, extremities trauma, neck and back trauma, chest trauma, pelvis trauma, burns, electric shock)
5. Obstetric and gynecologic emergencies (examination of the pregnant women and delivery, bleeding in pregnancy, hypertension in pregnancy, trauma in pregnancy, CPR of pregnant women, vaginal bleed)
6. Pediatric emergencies (pediatric emergency examination, pediatric trauma examination, pediatric burns, pediatric anaphylaxis and allergies, pediatric asthma, croup, pediatric convulsions, emergency pediatric glycemic conditions, pediatric overdose and intoxication, pediatric pain management, pediatric heart rhythm disorders)
7. Pediatric life support (basic pediatric life support, foreign body airway obstruction, advanced pediatric life support, newborn life support, death of a child)
8. Support for victim of abuse (suspected child abuse, suspected abuse of vulnerable adults and recognizing rape, sexual abuse)

The guidelines in the textbook are based on the UK Ambulance Service Clinical Practice Guidelines from 2006 and its revised versions from 2009 and 2010 and are adapted to Croatian context. They contain internationally recognized algorithms and approaches, such as: ABCDE assessment approach, AVPU consciousness scale, Glasgow Coma Scale, SAMPLE mnemonic acronym, descriptions of standardized physical examinations, measurement of vital parameters, recording of electrocardiograms, and basic life support (BLS), international trauma life support (ITLS), and advanced life support (ALS) algorithms.

The other three publications are:

- *Croatian Index for receiving an emergency call in the medical dispatch unit*, a Croatian adaptation of the Norwegian Index for Emergency Medical Assistance, consisting of 36 symptom-based criteria cards (Bošan-Kilibarda et al., 2011)

- *National guidelines for pre-hospital and hospital emergency medical services working with patients in need of palliative care* (Lončarek, 2015), a 52-page manual

*- Guidelines for pre-hospital and hospital emergency medical services in case of chemically related accidents* (Turk et al., 2018), a 153-page textbook.
